# Barriers to the Uptake of Cervical Cancer Screening and Treatment among Rural Women in Ghana

**DOI:** 10.1155/2019/6320938

**Published:** 2019-11-03

**Authors:** Charity Binka, Samuel H. Nyarko, Kofi Awusabo-Asare, David T. Doku

**Affiliations:** ^1^School of Public Service and Governance, Ghana Institute of Management and Public Administration, Achimota, Accra, Ghana; ^2^Department of Population and Behavioural Sciences, School of Public Health, University of Health and Allied Sciences (UHAS), Hohoe, Ghana; ^3^Department of Population and Health, University of Cape Coast, Cape Coast, Ghana

## Abstract

**Background:**

This study sought to explore the barriers to the uptake of cervical cancer screening and treatment in the North Tongu district of Ghana.

**Methods:**

Twenty-five in-depth interviews were conducted, while three focus group discussions were held among respondents. The data were analysed with the R package for qualitative data analysis using a thematic analytical approach.

**Results:**

Low level of knowledge about the disease and screening services, personal or psychological convictions, and cost of screening and treatment coupled with a low level of income were the barriers at the individual level. Perceived health personnel attitude, perceived lack of privacy, and misdiagnosis were the barriers at the institutional level while the sociocultural belief system of the communities about the etiology of the disease was the barrier at the community level. Inadequate education about the disease, lack of funding and access to screening facilities also constrained screening and treatment at the policy level.

**Conclusions:**

Cervical cancer screening and treatment are constrained at multiple levels in rural Ghana. This study underscores the need to address the low uptake of cervical cancer screening and treatment at the individual, community, institutional, and policy levels simultaneously.

## 1. Background

Cervical cancer was the second prevalent cancer but the leading cause of cancer deaths in Africa in 2018 [1]. It is estimated that by 2025, about 78,879 women living in Africa will be diagnosed with cervical cancer annually, while 61,671 will die from the disease [2]. Studies have shown that regular cervical cancer screening leads to early detection [3, 4]. According to Saslow et al. [5], cervical cancer screening should begin at age 21. Thus, women aged less than 21 should not be screened regardless of the age of sexual initiation or other behaviour-related risk factors. Furthermore, according to the World Health Organisation [6], screening for cervical cancer among women ages 30 and 49, for at least once, will reduce deaths from cervical cancer. Over the past 50 years, cervical cancer screening has been effective in reducing morbidity and mortality in high-income countries as against the situation in low-income countries, where effective cervical cancer screening programmes are virtually absent [7].

In Ghana, the Ministry of Health (MOH) and the Johns Hopkins Programme for International Education in Gynecology and Obstetrics (JHPIEGO) introduced cervical cancer screening and testing in the country in 2001 [8]. In 2004, the MOH officially incorporated cervical cancer screening into its National Reproductive Health Service Delivery Guidelines [9]. As part of this policy, human papillomavirus (HPV) vaccine and HPV DNA testing were licensed for use in public hospitals [10]. The Pap test and visual inspection with acetic acid (VIA) were the cervical cancer screening methods approved by the Ministry of Health of Ghana [11]. Nevertheless, the prevalence and mortalities due to cervical cancer are still high in Ghana [12].

Ghana has no systematic national cancer programme, and the development of a national cancer registry is at the undeveloped stage [8, 13]. In the absence of a national screening programme, most of the cervical cancer screenings that take place in the country can be described as opportunistic screening, where doctors request Pap smears or Visual inspection with acetic acid (VIA) for patients who are seen in clinics for either general medical examinations or for consultations unrelated to cervical cancer [13]. Cervical cancer prevention is, however, not commonly promoted and only a few women screen while infected women do not seek treatment [10, 14].

Findings from a few studies indicate that lack of knowledge about cervical cancer among Ghanaian women may be a barrier to cervical cancer screening and treatment [11, 14]. Analogously, other studies have also found that uptake of screening was low because of a lack of awareness about the disease and screening services [15, 16]. However, there is a dearth of in-depth but comprehensive information on factors that constrain the uptake of screening and treatment in the country. In this study, we explore the barriers that militate against the uptake of cervical cancer screening and treatment among women in a rural setting in Ghana.

## 2. Theoretical Framework

This study is guided by the socioecological model of McLeroy et al. [17] analogous to what has been done by Daley et al. [18]. McLeroy et al. [17] identify two key formed concepts: multiple levels (behaviour affects and is affected by multiple levels of influence) and reciprocal causation (individual behaviours shape and is shaped by the social environment). In this model, the patterned behaviour is considered as the outcome of interest and is viewed as being determined by intrapersonal, interpersonal, institutional, community, and public policy factors. An implicit assumption of these levels of analysis is that health promotion interventions are based on our beliefs, understandings, and theories of the determinants of behaviour and that these five levels of analysis reflect the range of strategies currently available for health promotion [17].

The intrapersonal factors include characteristics of the individual such as knowledge, attitude, behaviour, self-concept, and developmental history of the individual. The interpersonal factors refer to the interpersonal processes and primary groups (formal and informal social network and social support systems) such as the family, workgroup, and friendship networks [17]. The institutional factors have to do with the social institutions with organisational characteristics and formal (and informal) rules and regulations for an operation that constrain or promote behaviours. The community factors refer to the relationships among organisations, institutions, and informal networks among defined boundaries [17]. The public policy factors include local, state, and national laws and policies that affect behaviours [17]. These five levels of McLeroy et al.'s [17] model were adapted into four levels, by collapsing the intrapersonal and interpersonal levels into individual-level factors. The decision to aggregate the two levels was based on the emerging themes from the study. In this context, barriers to screening and treatment associated with the individual's personal characteristics such as knowledge and psychological factors among others were considered as individual-level factors. The institutional-level barriers also included those related to the attitudes of health service providers and the issue of confidentiality. Community-level barriers also captured issues concerning the belief system of the communities while the policy-level barriers comprised the actions and inactions of government policies in the country. This model was used to guide the study because it provides an opportunity to look at the barriers to cervical cancer screening and treatment from a hierarchical perspective. This approach is expected to provide the opportunity for policymakers to tackle the situation at multiple levels rather than solely at the individual or the national level.

## 3. Materials and Methods

### 3.1. Study Area and Design

The study area for this research was the Battor community including the Battor Catholic Hospital in the North Tongu District of the Volta Region, Ghana. This community was selected because it houses one of the few health facilities that provide cervical cancer screening and treatment in the country. A qualitative study was conducted in order to explore and understand the subjective motives or reasons and experiences of respondents in terms of factors that militate against screening uptake in the community.

### 3.2. Study Population

The study population constituted two main categories of women in the study setting. The first included cervical cancer patients who attended the gynecology unit of the Battor Catholic Hospital in the North Tongu District, Volta Region, Ghana. The second category included women aged between 30 and 65 living in and around the surroundings of Battor who were registered at the Battor Catholic Hospital but have not screened for cervical cancer before.

### 3.3. Sampling Procedure

In selecting the respondents, 60 cervical cancer patients were identified from the gynecological department registry of the hospital at the time of the study. However, only 15 were available and willing to participate in the study. This was because a considerable number of them had died while some could not be reached because of wrong telephone numbers and some were unwilling to participate. The women who consented were contacted through their telephones and then interview times were scheduled for in-depth interviews. Also, 10 women who had never screened for cervical cancer were conveniently selected based on thematic saturation from the hospital setting to participate in the study.

In addition, a total number of 30 women were conveniently selected for three different focus group discussions comprising 10 women in each group. Two of these discussions were held in a nearby community called Kekpo while the third discussion was held in the hospital setting. The younger groups were then separated from the older group in order to provide a free and convenient atmosphere for all the respondents to equally and effectively participate in the discussions.

### 3.4. Data Collection Procedure

In-depth interviews were conducted for the cervical cancer patients and the women selected from the hospital who had never screened using an in-depth interview guide. Also, focus group discussions were conducted among three different groups of women from the Battor community. The in-depth interview and focus group discussion guides included issues on the background characteristics of respondents, as well as various levels of factors that constrain cervical cancer screening in the community. The interviews and discussions were conducted in three Ghanaian languages, namely, Ewe, Twi, and Dangme. Three female research assistants who are fluent in these languages were recruited and trained for the study. The interviews and discussions were tape-recorded and then peer-reviewed by the research team during the process in order to ensure data quality. Prior to the study, institutional approval was sought from the Battor Catholic Hospital while ethical clearance was obtained from the Ghana Health Service Ethics Review Committee (GHS-ERC). Written informed consent was also obtained from the respondents before participation.

### 3.5. Analytic Procedure

The tape-recorded interviews and discussions were transcribed in English. These were done by research assistants who were competent in Ewe, Twi, and Dangme local languages as well as in the English language. The tape-recordings together with the transcripts were then forwarded to experts in these local languages to review and revise the content in order to ensure validity. The data were processed with the R programming language package for Qualitative Data Analysis (RQDA) (version R-3.2.2). The transcripts were then coded and analysed using the thematic approach to qualitative data analysis as well as the deductive approach which helped to generate the deductive codes for the emerged themes from the data. The results were presented in the form of selected quotes from the respondents and used to support the contextual analysis and discussion of the findings.

## 4. Results

### 4.1. Background Characteristics of Respondents

A summary of the background characteristics of respondents has been presented in Table 1. The majority (10) of the 15 cervical cancer patients were aged 50 and above. Also, the majority (10) had at least secondary education or were married while the remaining respondents were either separated, divorced, or widowed. The majority were also self-employed and had about 4 to 6 children. The respondents who had never screened for cervical cancer comprised women aged 31–46. For the three focus group discussions, respondents for the first and second discussions comprised women aged 35–45, whereas the respondents for the third discussion comprised older women aged 46–65.

### 4.2. Individual-Level Barriers

The individual-level barriers to screening generally included low awareness of screening and screening facilities, personal factors, screening procedure, and low income. Knowledge about cervical cancer and where one could obtain a screening service is quite important to the uptake of screening and treatment of the disease. The study showed low awareness of screening services as a major barrier to the uptake of cervical cancer screening. Some of the respondents explained that they had not received cervical cancer screening information and therefore they were unaware of any available cervical cancer screening services. This could be deduced from the following statement by a respondent:

*“I have heard of the disease on the radio, but I have not heard of the screening that is why I have not screened.”* (Respondent who had never screened, 41 years)

Some respondents were also not aware of the few health facilities providing cervical cancer screening services in the country. A statement made by a respondent confirmed this:

*“I am not aware of any facility that conducts screening for cervical cancer.”* (Respondent who had never screened, 46 years)

A number of personal or psychological factors also constrained respondents' decision to go for screening. Among those who had knowledge of the importance of screening, fear of diagnosis and perceived fatalism associated with the disease were commonly expressed as factors militating against the uptake of screening and treatment services. This concern was expressed by women who did not have the disease but had never screened. They explained that they kept on postponing screening for fear of the outcome of the result. The following statement was made by one respondent:

*“Fear is what holds us back from undergoing a test for cervical cancer. There was such a test some time ago. Some people were diagnosed, and the disease was removed from their bodies. Those who were afraid refused to go for the screening.”* (FGD 1)

To some respondents, the screening procedure was their challenge. The respondents perceived the screening as a painful procedure that involved inserting metal into the vagina of a woman. They explained that those who had ever gone through the process had told them of their painful experiences. A respondent in a focus group discussion noted that:

*“The reason why some of us refuse to go for screening is about the reports we have from those who had already attended the screening services in the hospital. We heard that they have to open up their vagina for a metal to be inserted during the screening process. We were told that the screening was quite a painful experience.”* (FDG 2)

Another barrier to screening was shyness and embarrassment on the part of the respondents. This was mentioned in focus group discussions and in-depth interviews involving female respondents. The respondents said that being examined by a male health practitioner would be particularly embarrassing. Some women also reported that they would be embarrassed no matter the sex of the health personnel. This was a major barrier, especially for older women. A response from an in-depth interview emphasises this:

*“For me, I feel shy to open that place for anybody...even for a woman because that place is private to you and your husband*. *The doctors should find an alternative test or they can use a blood test.”* (Respondent who had never screened, 43 years old)

Low level of income was observed as one of the factors that restrained women from screening for cervical cancer. Some respondents explained that poverty prevented them from going to screen. One respondent in an in-depth interview reported:

*“For now, I am not working. If I had a job, I would have paid for the cost of the test. There are some women who had no money, transport fare, or health insurance and because of that they could not go for screening.”* (Respondent who had never screened, 41 years old)

Treatment of cervical cancer is expensive and puts a heavy financial burden on patients and their families. Some patients reported spending about US$ 1500 or more for surgery or other forms of treatment, depending on where the service is acquired. Partners of some cervical cancer patients reported that the expenses associated with the treatment of their wives actually strained them and the entire family financially. This is because some partners and their wives were mainly peasant farmers with low income. This has brought financial challenges to them, leading to a delay in seeking treatment.

### 4.3. Institutional-Level Barriers

Some institutional-level factors also appeared in the results as barriers to the uptake of cervical cancer screening and treatment. These included concerns about the privacy issues of patients seeking healthcare, health worker attitude toward patients, and perceived misdiagnosis of health conditions by health personnel. Some respondents who had not screened before also raised concerns about not being assured of their privacy and the attitude of some health personnel during screening and treatment services as deterrents to a hospital visit for screening. The respondents said they may not go for screening if they did not trust that the health personnel would keep their information confidential. One respondent reported:

*“If I do not get privacy in the room where the test will be done and if the health workers are not friendly, or if they do not explain things better to me, I might abstain from doing it.”* (Respondent who had never screened, 37 years old)

Some respondents were also concerned about the hostile attitude of the health workers. They explained that the unfriendly attitude of some health personnel is a barrier to their uptake of the screening service. One respondent said:

*“If health providers are not friendly...I am not sure I will go there for screening.”* (Respondent who had never screened, 31 years old)

Some respondents were also worried about possible misdiagnosis by the health personnel, which they believed may cause them unnecessary stress or worsen their health condition. Their concerns were based on previous experiences where they were given false results when they went to screen or seek treatment for various conditions. One woman who had not screened for cervical cancer before reported that she would not go for any screening programme because of an earlier instance where her health condition was misdiagnosed. She reported:

*“I have told myself I will never go for any screening programme because one day I went to the hospital with my husband who was then sick and coughing. They ran a test to confirm it was tuberculosis. They advised that I come with all my children to be screened and I was then misdiagnosed with tuberculosis. On another occasion, one of my children was sick and we were all asked to screen, and I was misdiagnosed with sickle cell disease. So, when it comes to screening, I decided not to go. Because, whenever I went, the results were frightening, which were not even true.”* (Respondent who had never screened, 43 years old)

### 4.4. Community-Level Barriers

The community-level barriers were based on sociocultural factors. These had to do with belief systems or religion, use of traditional medicine, and gender relations that usually prevail in the rural communities. Some respondents did not want to be associated with cervical cancer because of the belief that the disease was caused by a promiscuous lifestyle. Some thought cervical cancer was a punishment from their Gods for an offense. For this reason, they prefer to go for divine intervention instead of going for screening or treatment at the hospital. Consequently, some women rather prefer to seek remedy from churches and spiritual healing centres. Some respondents indicated that churches prescribed a number of days of fasting and prayers and making some special offerings to the church. One cervical cancer patient said:

“....*I have been watching Emmanuel TV programme and I decided to go to T.B Joshua in Nigeria and add prayers because God is the overseer of everything. With cancer, you cannot just treat it like that. You have to treat it with God because God is the healer. That man, I know very well, is a healer.”* (Cervical cancer patient, 43 years old)

Another sociocultural barrier identified from the study was the belief in traditional medicine. Even though some acknowledged the importance of going to the hospital, they believed traditional medication had to be sought first. They rather seek help from local herbalists first before going to the hospital as a last resort. The use of local herbs or medicine stemmed from the belief that traditional medicine was more potent for the treatment of such a disease. One cervical cancer patient in an in-depth interview replied:

*“...When the bleeding became unbearable, I started using local medicines. After taking the local medicine for some time, the condition became worse. The blood flow became more and more and started clotting. So, it was uncontrollable that I had to finally seek treatment from the hospital.”* (Cervical cancer patient, 52 years old)

Another barrier that emerged from the study was normative gender relations, where men assumed the role of decision-makers even when it had to do with the health of women. Consequently, some respondents explained that they needed the approval of their partners to undergo screening for the disease. A woman in a focus group discussion said:

*“Traditionally, no other man should touch the private part of any married woman. So, for us women, we need to get our husband's approval first.”* (FDG 3)

Related to the above was women's reliance on male partners for various supports including financial support. Some of the respondents who had never screened reported that they could not go for screening without the financial support of their husbands. This is because they felt their husbands should be responsible for the cost of screening and treatment. The following was what a respondent said:

*“Screening involves money and my husband is the only one who can extend that kind of financial help to me.”* (Woman who had never screened, 33 years old)

It is, therefore, a cultural norm that married men are expected to bear the cost of health care of their wives and their children. In view of this, even though some women could afford to pay for the cost of screening and treatment, they still depended on their husbands for support. Consequently, if the men delay or fail to financially support them, it becomes a challenge to the health-seeking behaviour of the women.

### 4.5. Policy-Level Barriers

At the policy level, the study identified a number of barriers such as lack of policies on the management of the disease or poor implementation of existing ones. Other barriers were low education on cervical cancer screening and treatment and inadequate screening and treatment facilities. Respondents who had never screened explained that there was low awareness creation on the disease in the communities. They further explained that as a result of this, some of them had little or no knowledge about the disease. This prevented many women in the communities from screening. Some respondents reported that they would have gone for screening if they had received adequate education or information on the disease. The following statement was made by a respondent:

*“Well, I do not think awareness had been created enough about the disease and that is the problem. Women would not come voluntarily to tell you they have heard of any screening and they want to do it. They should educate us enough through the media and other ways of education and then we can go for it.*” (Respondent who had never screened, 37 years)

Another policy issue identified as a barrier to the uptake of cervical cancer screening and treatment was the lack of government subsidy for the screening and treatment services. Some respondents reported that even though they were aware of the disease, they could not afford the cost of screening and treatment. They noted that the government could have subsidised the cost of screening and treatment or even made it free for every woman in the communities to access the services. One respondent said:

*“Some of us cannot pay for the cost. Because they asked us to pay, they end up getting a few people taking part, not everyone can get money to test for it. But if the screening and some of the treatments are for free, a lot of us will get involved. If a patient is put on treatment and recovers, she can become useful to the country. Therefore, the government should regularly help everyone for them to take part in the screening.*” (Respondent who had never screened, 37 years old)

Unavailability or inaccessibility of screening and treatment facilities had also emerged as a factor militating against the uptake of screening and treatment for the disease among women in the study communities. Some respondents reported that there was no screening and treatment facility in their neighborhood since they were coming from quite a remote area. They explained that the long distance to health facilities made it difficult for them to go for screening and treatment. Some respondents said if more screening and treatment facilities could be built close to where they lived, it would help in improving uptake of screening and treatment. The following statement from a respondent explains this further:

*“Some of us are coming from a rural area where there are no health facilities. We have to come this far before we can come and screen. If only the government can provide more health facilities, it will promote the uptake of cervical cancer screening and treatment in our communities.”* (Respondent who had never screened, 46 years old)

## 5. Discussion

This study explores the barriers to the uptake of cervical cancer screening and treatment in the North Tongu District of Ghana, using the socioecological model as a guide. The study has found multilevel barriers that constrain cervical cancer screening and treatment at the individual, community, institutional, and policy levels. The individual-level factors include low awareness of screening, personal factors, screening procedure, and low income while institutional factors include privacy issues, health worker attitude, and perceived misdiagnosis. Community-level factors comprise the belief system use of traditional medicine and gender relations, whereas policy factors have to do with low education on cervical cancer screening, lack of government funding, and screening facilities.

At the individual level, the uptake of cervical cancer screening among the women was constrained by the low level of knowledge about the availability of cervical cancer screening services and where to get them. Most of the women pointed out that they initially did not know about screening opportunities and that was one of the reasons why they could not screen for the disease. Individual-level barriers to preventive health and screening, in particular, have been reported in previous studies [19–21]. A number of studies have come out with similar findings. For instance, it was established that in the low and middle-income countries, especially in rural areas, knowledge and awareness of cervical cancer screening are very low, and this is one of the main factors that constrain women's uptake of cervical cancer screening initiatives [19–21]. This implies a lack of or low level of education and sensitisation on the disease among respondents.

Furthermore, the fear of screening itself, screening outcomes, diagnostic procedures, and treatment played a major role in preventing women from cervical cancer screening [22]. From their experiences, some women feared misdiagnosis of the disease and these experiences act as factors militating against screening for the disease. The case of perceived pain and anxiety associated with the screening and treatment procedures also served as a constraint to going for screening, as women believed that the insertion of screening instruments into their vagina orifice could be quite painful and unbearable. This may have implications for providing more convenient ways of screening.

It also emerged that some women, particularly the older ones, are shy of or embarrassed by exposing their nakedness to especially male health personnel when they go for screening. This has consistently emerged in several studies where shyness, uneasiness concerning medical examination, and feeling ashamed to expose private part lead to nonscreening particularly when the screening is being done by male health personnel [23]. The findings further show economic constraints as some of the factors hindering cervical cancer screening and treatment among women in the study setting. Evidence from several studies confirm that economic factors, particularly low income or high cost of screening and treatment are a major barrier to screening and early diagnosis of the disease. For instance, Williams and Amoateng [14], Basu et al. [22], and Keshavarz et al. [23] have all provided evidence of the role played by socioeconomic constraints against early screening and treatment for the disease.

In this study, issues concerning the costs of screening and treatment have been raised by cervical cancer patients as well as their partners. Poverty was cited by some of the respondents as the reason why women could not go for screening and treatment of the disease. This may be the result of the fact that the respondents are mainly unemployed, and even those who are employed are only self-employed in petty trades and are, therefore, earning very little to afford the cost of screening and treatment charged by the health facilities. Nevertheless, it is believed that economic empowerment alone does not guarantee the uptake of cervical cancer screening. Thus, a combination of economic issues and other factors such as knowledge and perceived seriousness of the disease also influence screening uptake either by delaying the uptake or preventing it on the whole [24].

Moreover, this study finds that institutional-level barriers constrain the screening and treatment of the disease. The attitude of health personnel and the issue of confidentiality with medical information and privacy are found to be some of the factors militating against screening for the disease. This is consistent with the findings of a number of previous studies [21, 25]. Fort et al. [21] and Mutyaba et al. [25] have established that inability to maintain the confidentiality of test results, rude behaviour of some health personnel, and concerns about privacy during screening serve as barriers to the uptake of screening services. Some women consider the unfriendly attitude of some health personnel and concerns about medical information privacy as crucial factors in determining whether or not they would go for cervical cancer screening and treatment. These concerns may likely serve as barriers to the uptake of screening and treatment and in turn have negative implications for the fight against the disease.

Additionally, at the community level, sociocultural factors which have to do with the belief system of the study communities hindered the uptake of screening and treatment among the respondents. The belief that cervical cancer is caused by retribution from God or the deities of their land wrongdoings or that the disease is as a result of a curse from sexual promiscuity may constrain their propensity to seek for screening services. To some, being diagnosed with cervical cancer means that the woman was unfaithful to her spouse or immoral. Consequently, some women hide their condition or resort to alternative solutions including divine interventions at churches and traditionalists as well as traditional medicine, which may delay or constrain seeking screening and appropriate treatment services for the disease.

Some issues of normative gender relations also emerged as barriers to screening and treatment. Most women need to seek approval and financial support from their partners before they could seek treatment for any particular disease. Thus, some of the respondents who were married could not take initiative concerning their health, as they had to depend on the support of their partner to take part in cervical cancer screening, diagnosis, or treatment. In some previous studies, it was observed that some women find it very difficult to convince their partners for social or financial support to visit a healthcare facility for screening, especially if they are not visibly ill [21, 23, 26]. Therefore, their attendance of screening may likely depend on the approval of male partners and their willingness to support the women financially.

As well, this study provides evidence that inadequate efforts on public education about the disease serve as a policy-level barrier to screening and treatment of the disease. The respondents explained the fact that there is little or no sensitisation on the disease in the local media or other channels within the health system. A similar finding has been observed by Daley et al. [18], which they argue that improving education on cervical cancer screening is effective in increasing knowledge on cervical cancer and increasing screening uptake. Lack of subsidy or funding for the cost of screening and treatment by the government is also a challenge to screening uptake. Daley et al. [18] also identified funding as one of the factors constraining screening and treatment of the disease. Respondents, therefore, explained that subsidising the cost of screening and treatment to make it affordable or free will motivate them to go for screening and treatment for the disease.

The study further finds that inaccessibility or unavailability of screening and treatment facilities in the study communities is a challenge to screening and treatment uptake in the study setting. According to Daley et al. [18], the isolation of rural areas, both geographically and resource-wise, is a substantial obstacle in providing preventive care in terms of cervical cancer. A similar finding has also been established by Tung et al. [27] in their study. It has been found that screening and treatment services are unavailable in the study communities and the only available one is quite remote from these communities. From this perspective, developing effective and comprehensive government policy for cervical cancer screening and treatment has crucial implications for the fight against the disease in Ghana.

## 6. Conclusions

The uptake of cervical cancer screening and treatment is constrained by different categories of barriers at the individual, institutional, community, and public policy levels in Ghana. These range from the level of knowledge about the disease to lack of funding for screening and treatment services as well as gender relations. This provides concrete evidence for the use of multilevel strategies in the promotion of the uptake of cervical cancer screening and treatment in the study setting and in Ghana, at large. In effect, it becomes imperative to intensify public education about the disease in the study setting. This can be done through frequent radio or television programmes and through frequent local women seminars on the disease in the local language. This will likely enlighten the local women on the disease and, in turn, encourage them to go for screening for the disease.

The government of Ghana can also subsidise the cost of screening and comprehensive and treatment of the disease for women in the locality through a systematic and comprehensive national cervical cancer screening programme while expanding the provision of screening and screening and treatment services to other health facilities in the communities in the district. This will make screening and treatment affordable for women who cannot afford the perceived high cost of screening and treatment. Additionally, screening and treatment services should be accessible to encourage patronage by women. As a preventive measure, screening for cervical cancer could be made part of the services provided under the National Health Insurance Scheme of Ghana and made mandatory for all women under the scheme. Health facilities should also provide counseling units that could support women and allay their fears surrounding the screening and treatment procedures.

## Figures and Tables

**Table 1:** Summary of respondents' background characteristics.

Cervical cancer patients	Number of respondents
Age group	
30–39	1
40–49	4
50+	10
Education	
None	1
Primary	4
Secondary/higher	10
Marital status	
Married	10
Separated/divorced/widowed	5
Employment status	
Unemployed	5
Self-employed	10
Number of children	
1–3	3
4–6	9
7+	3
Women who had never screened (31–46 years)	10
Focus group discussion	
FDG 1 (35–45 years)	10
FDG 2 (35–45 years)	10
FDG 3 (46–65 years)	10

## Data Availability

The dataset used to support the findings of this study is contained within the article.
